# A Framework for Optimizing Deep Learning-Based Lane Detection and Steering for Autonomous Driving

**DOI:** 10.3390/s24248099

**Published:** 2024-12-19

**Authors:** Daniel Yordanov, Ashim Chakraborty, Md Mahmudul Hasan, Silvia Cirstea

**Affiliations:** School of Computing and Information Science, Anglia Ruskin University, Cambridge CB1 1PT, UK; dv.yordanov01@gmail.com (D.Y.); md.hasan@aru.ac.uk (M.M.H.); silvia.cirstea@aru.ac.uk (S.C.)

**Keywords:** autonomous steering, deep learning, Unity3D

## Abstract

Improving the ability of autonomous vehicles to accurately identify and follow lanes in various contexts is crucial. This project aims to provide a novel framework for optimizing a self-driving vehicle that can detect lanes and steer accordingly. A virtual sandbox environment was developed in Unity3D that provides a semi-automated procedural road and driving generation framework for a variety of road scenarios. Four types of segments replicate actual driving situations by directing the car using strategically positioned waypoints. A training dataset thus generated was used to train a behavioral driving model that employs a convolutional neural network to detect the lane and ensure that the car steers autonomously to remain within lane boundaries. The model was evaluated on real-world driving footage from Comma.ai, exhibiting an autonomy of 77% in low challenge road conditions and of 66% on roads with sharper turns.

## 1. Introduction

Autonomous driving technology is maturing progressively and at an increasing pace. By 2035, 40% of new cars in the UK will have self-driving capabilities, and many public services, such as transportation and supermarket delivery, will be run by self-driving vehicles, as estimated in the UK Government Report [1]. By ensuring that the algorithms controlling a vehicle would be quicker and more accurate at making decisions, the combined result of technological advancements in the fields of computer vision and artificial intelligence are put into the automotive industry and are promising to decrease fatality rates by up to 90% [2]. Numerous additional benefits, including commutes that are quicker and more enjoyable as well as cleaner and greener cities, are foreseen as a result of less emissions contributed to ride-sharing ideas [3].

An autonomous vehicle (AV) is defined as a vehicle that combines a variety of sensors in order to perceive and navigate its environment without any human intervention [4]. The Society of Automotive Engineers divides autonomous driving into six levels of autonomy from manual operation (Level 0) to no human monitoring (Level 6) in the J3016_202104 standard [5]. As of today, Honda has introduced to the market, approved by the law, Level 3 autonomous vehicles [6], while transport providers have started introducing autonomous taxis in two states in the USA [7]. However, lane detection and automatic steering remain challenging parts of achieving higher autonomy in the transport industry. Having the ability to detect the lane boundaries of the road in non-homogeneous conditions and stay between them is one of the fundamentals of developing an AV and safely navigating public roads. While machine vision and deep learning algorithms show promising ability to achieve safe navigation in Avs [8], the limited availability of driving data that comprehensively cover a variety of road configurations and traffic conditions continues to be a barrier to optimizing autonomous driving.

This paper proposes a new framework for optimizing lane detection and steering based on the automated generation of deep learning training scenarios and a convolutional neural network (CNN). A virtual reality sandbox environment was developed in Unity3D (version 2021.3.16f1) to automatically generate driving scenarios and extract data related to lane boundaries, steering angle, and car position within the lane. This environment features a variety of road textures, road configurations, and lane markings, and can be customized as needed, including with lighting and weather conditions. A data selection algorithm is applied to ensure the selection of a balanced training dataset and avoid overfitting. The data thus selected are pre-processed to suppress noise and to further expand the training set through augmentation techniques. A CNN model is proposed and trained using the pre-processed dataset to predict steering angles based on input road images. Once the model has undergone training and its performance has been validated within a controlled environment, it is then evaluated on real-world driving footage to assess its autonomy.

The main contributions of this work are (i) an end-to-end customizable framework for training an automated lane detection and steering system on multiple driving scenarios; (ii) implementation of a behavioral driving model where lane markings are not necessary to detect the lane; (iii) automated frame annotation with the corresponding steering angle.

## 2. Background

A number of authors have proposed autonomous lane detection systems using computer vision [9,10,11,12]. Ref. [13] implemented curve modeling and convolution-based features eliminating occlusions and predicting a variable number of lines. Their model integrated Bezier curves into deep learning and applied feature flip fusion to identify symmetrical curve models of the two adjacent lines. Ref. [14] devised a real-time method called LaneATT that effectively detects lane patterns using an anchor-based mechanism that combines global information to achieve more efficient lane matching, particularly in challenging scenarios involving occlusion and missing lane markers. Ref. [15] proposed a method treating lane detection as a row-based selection problem with a global feature. They addressed the computational cost and challenges of previous techniques, like fast pipeline self-attention distillation (SAD), by introducing a structural loss that incorporates prior lane information to enhance computational speed. Their approach selects lane positions at predefined image rows using global features. Ref. [16] converted road images from RGB to HLS followed by a color segmentation technique and thresholding method to identify the lane marking painted in white or yellow. To detect the yellow and white lane lines and curves in a complex environment, Ref. [17] utilized a new lane detection technique that incorporates the processing of yellow lane lines in HSV color space and white lane lines in grayscale space followed by canny edge detection, inverse perspective transformation, and sliding window polynomial fitting in order to achieve real-time lane detection. Combining a ridge detector and regional Gaussian distribution random sample consensus (G-RANSAC) for road lane detection, Ref. [18] proposed that efficiently captures detailed road lane characteristics from aerial-view images, and extracts lane features using the ridge detector. By leveraging the advantages of model-based and feature-based detection methods, their approach achieves superior performance across diverse testing scenarios. Computer vision methods are able to generate enhanced accuracy as they easily identify lane patterns and can be continuously improved; however, they may face challenges when real-world conditions do not align with the model’s assumptions [19].

When the convolutional neural network (CNN) AlexNet achieved a top five error in the ImageNet Large-Scale Visual Recognition Challenge (ILSVRC), it marked a significant milestone for deep learning algorithms [20,21,22]. Since then, deep learning has emerged as a promising tool for lane detection. Recent techniques include end-to-end segmentation-based methods such as GCN [23] and SCNN [24], as well as GAN-based methods like EL-GAN [25]. Knowledge distillation [26] and attention maps [27] have also introduced new ideas for lane detection, exemplified by approaches such as structure-aware detection SALMNet [28]. DAGMapper [29] offers a valuable perspective by explaining the structure of lane lines through directed acyclic graphs.

Ref. [30] developed a new 1D signal-based deep learning method for road detection, which surpasses traditional model-based approaches by effectively handling difficult conditions such as rain and shadows, resulting in superior performance. However, this type of deep learning approach necessitates a high-capacity platform, which poses limitations on its utilization in embedded systems [19].

Ref. [31] developed LaneNet, a deep neural network approach for lane line detection. The process involves two stages: lane edge proposal and lane line localization implementing a lane edge proposal network. While this model focuses on lane line detection and may have limitations in detecting other road signs, it offers high running speed, low computational cost, and robust performance in autonomous vehicle-based systems. Similarly, by modifying and optimizing the feature extraction and decoder modules of an existing LaneNet model, Ref. [32] introduced a novel deep-learning framework that reduced computational costs and achieved one of the enhanced real-time performances enabling a reduction in post-processing resources. Ref. [33] proposed a deep-learning approach that offers a real-time end-to-end system for lane boundary identification, clustering, and classification, incorporating two cascaded convolutional neural networks and achieving a notable high-performance score while maintaining real-time computational speed. Combining computer vision and AI algorithms, Ref. [34] utilized an amalgamation of HOG features and convolutional neural networks (CNNs) to extract features, identify objects, and perform semantic segmentation in the context of automated driving. They employed fully convolutional networks (FCNs) for this purpose. Additionally, they utilized relationship extraction techniques to estimate distances by analyzing the relationships between objects, including vehicle distance and speed. Furthermore, for event detection, they analyzed dynamic changes in targets, such as vehicle collisions and pedestrian crossings. On the other hand, Ref. [35] assesses the resilience of three-lane detection methods—camera computer vision (CV), chip-enabled raised pavement markers (CERPMs), and radar retroreflectors (RRs)—in the face of hazards. Employing the CARLA simulator, they utilized resilience engineering (RE) metrics to evaluate performance. Their findings indicate that infrastructure-based sensors exhibit greater resilience compared to conventional computer vision methods when exposed to hazardous scenarios.

The primary hurdle that current methods in lane detection encounter is the limited capacity for generalization [19]. Overcoming this limitation remains a significant challenge in the field. To overcome this challenge, Ref. [36] proposed CondLaneNet, a deep learning method for lane detection that detects lane instances and predicts their shapes dynamically. By employing conditional convolution and row-wise formulation, the model achieves discrimination at the instance level, as well as handling complex lane topologies and achieving enhanced outcomes on popular datasets. Similarly, Ref. [37] proposed a model by separating lane representation components through exploratory oracle tests. They then utilized an anchor-based detector to predict lane locations and confidence ratings, assessing intersection-over-union (IoU) with ground truth.

Despite the successful outcomes obtained so far, challenges caused by imbalance and redundancy in training data and by label fluctuation, especially when there are no visual markings on the road, hamper the performance of self-driving solutions.

## 3. Materials and Methods

**Figure 1:**
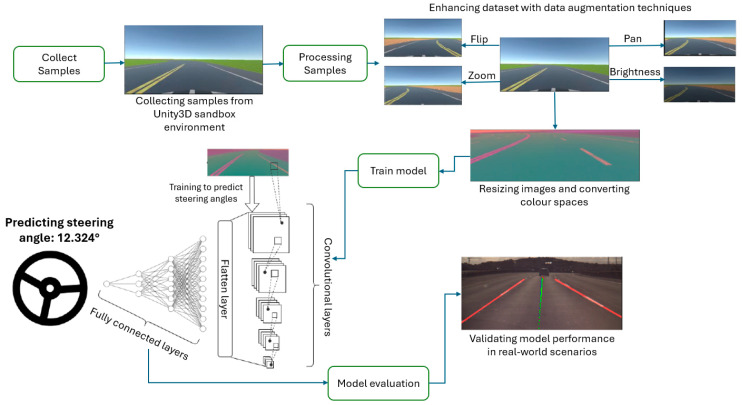
Step-by-step methodology of the project. (In the validation image, red lines represent detected lane boundaries, the blue line shows the road’s central alignment, and the green line indicates the vehicle’s projected path).

In this work, we address these challenges by proposing a framework for the automatic generation and annotation of driving data for a customizable variety of road scenarios and conditions. Automatic annotation ensures labels are consistent on the training data; a data selection algorithm reduces data redundancy from straight roads and stationary vehicles. A CNN-based behavioral driving model is implemented which does not require road markings to detect and steer within the lane. The framework we propose is suitable for “behavioral cloning” where an AI model attempts to learn an action by studying patterns in the way an expert performs the activity the model is required to clone [38]. Figure 1 illustrates the overarching methodology adopted for this project, commencing with the automated sample gathering process in a virtual Unity3D sandbox, followed by preprocessing of the collected data. Subsequently, a CNN model was devised and trained utilizing the preprocessed dataset. Finally, the efficacy of the model was evaluated through testing with data obtained from real-world scenarios.

### 3.1. Framework for Generating Driving Data

The initial phase of training a lane detection model entails the sample-gathering process. Manual collection of driving samples, e.g., from real-world driving footage, is a labor-intensive endeavor that requires massive annotation effort and is prone to (i) error as the steering angle can only be approximated for a frame and (ii) bias, as real-world driving data are inherently limited by the roads and geography of the place of recording. To alleviate this problem, a sandbox environment was developed in Unity3D to generate training samples by driving a virtual car fitted with one camera positioned on the front windshield, focused straight ahead to capture images of the turn. The quality of these samples holds paramount importance for the efficacy of the trained model and two innovations are proposed to increase the size, diversity, and accuracy of the generated samples: (a) diversify the road conditions; (b) automate driving within the virtual scenario.

#### 3.1.1. Diversification of Road Conditions

**Figure 2:**
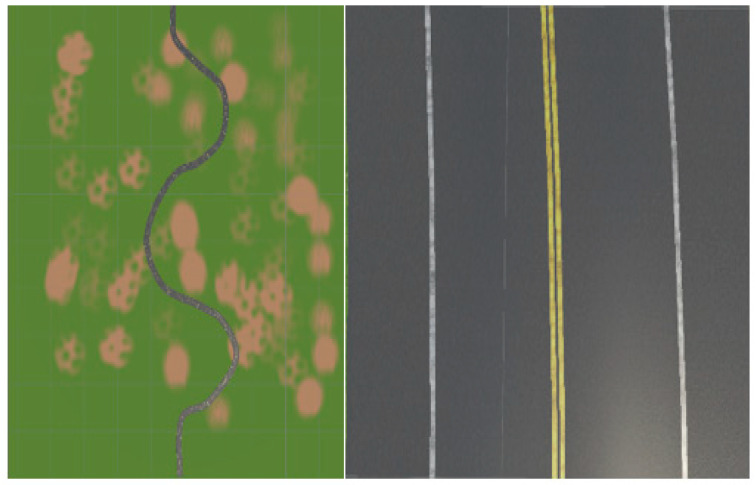
Depiction of a Chunk 1 driving scenario with multiple lanes and driving in both directions.

The proposed solution involves the development of a semi-automated procedural road generation framework in Unity3D using Kajaman Roads [39]. This approach allows pinpointing areas where the model encounters difficulties, such as negotiating sharp turns or traversing roads lacking lane markings. As seen in Figure 2, road segments tailored to address these specific challenges can be seamlessly integrated into the training environment. Moreover, the system incorporates a mechanism whereby the model identifies impending road terminations and dynamically loads subsequent segments, potentially selected at random, to enrich the sample diversity and enhance model robustness.

To enhance dataset diversity and the robustness of the model, a systematic approach was undertaken, resulting in the creation of four distinct chunks, each endowed with unique properties mirroring real-world driving scenarios. The first chunk, herein referred to as Chunk 1, is characterized by a single-carriageway configuration featuring one lane in each direction. Within this setting, the vehicle operates on the right-hand side of the road, adhering to a predefined trajectory situated at the midpoint of the lane.

As seen in Figure 2 the rationale behind crafting Chunk 1 lies in replicating a common driving scenario encountered in real-world environments, wherein roads typically comprise lanes accommodating traffic flow in both directions. By constraining the vehicle to navigate along the right side of the road, consistent with traffic norms in numerous jurisdictions, and adhering to a centralized path within the lane, the dataset encapsulates fundamental driving behaviors essential for autonomous vehicle navigation.

**Figure 3:**
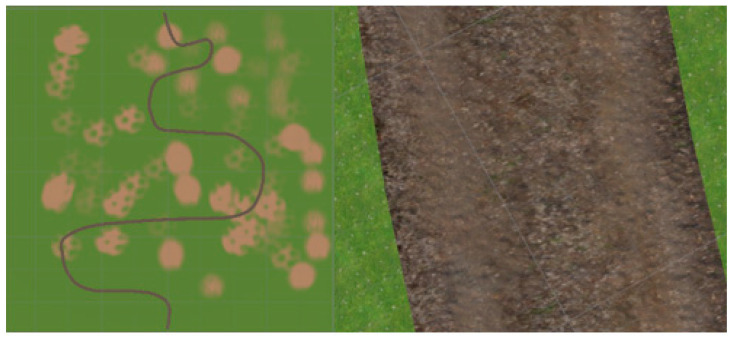
Road scenario with ambiguous lane demarcations (Chunk 2).

The conceptualization of Chunk 2 stemmed from the recognition of the ubiquity of roads devoid of clear lane demarcations in real-world driving contexts. Environments such as unmarked muddy roads pose unique challenges for autonomous vehicle navigation, necessitating models capable of robust performance under diverse and often imperfect roadway conditions, as seen in Figure 3. By incorporating this segment into the dataset, the model is exposed to scenarios reflective of the inherent variability and unpredictability characteristic of real-world driving environments.

**Figure 4:**
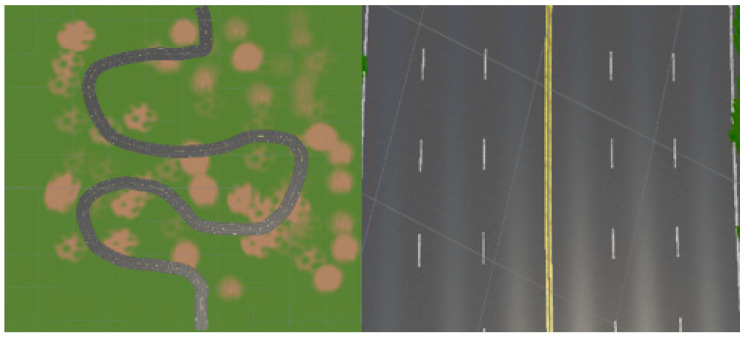
Highway environment featuring three lanes in each direction (Chunk 3).

As part of the dataset diversification strategy, Chunk 3 was formulated to encapsulate a highway setting characterized by three lanes in each direction, depicted in Figure 4. Within this scenario, the vehicle operates within the middle lane, simulating common highway driving conditions encountered in real-world environments.

**Figure 5:**
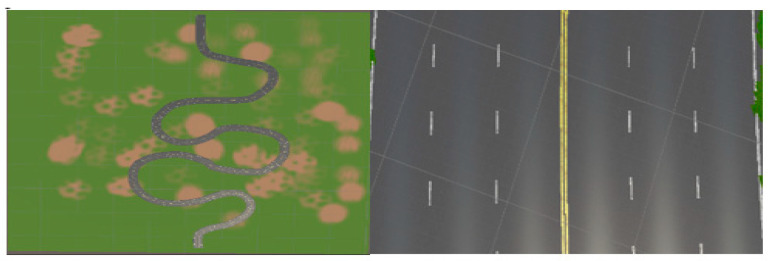
Chunk 4—A driving scenario on a three-lane highway environment similar to Chunk 3. The vehicle travels in the innermost lane, encountering diverse turn angles to enrich model training complexity and the diversity of the driving experience.

Chunk 4 of the dataset is designed to emulate a highway environment akin to Chunk 3, featuring a roadway with three lanes in each direction illustrated in Figure 5. However, in contrast to Chunk 3 where the vehicle navigates the middle lane, in Chunk 4, the vehicle traverses the innermost lane, situated closer to inbound traffic. Moreover, varying turn angles are incorporated into this scenario to introduce additional complexity and diversify the driving experience encountered by the model. The scenarios generated in the sandbox can be further diversified using Unity3D assets to produce varying lighting conditions (e.g., day, night) and weather effects (rain, fog) that affect visibility [40].

#### 3.1.2. Automating Driving for Data Collection

**Figure 6:**
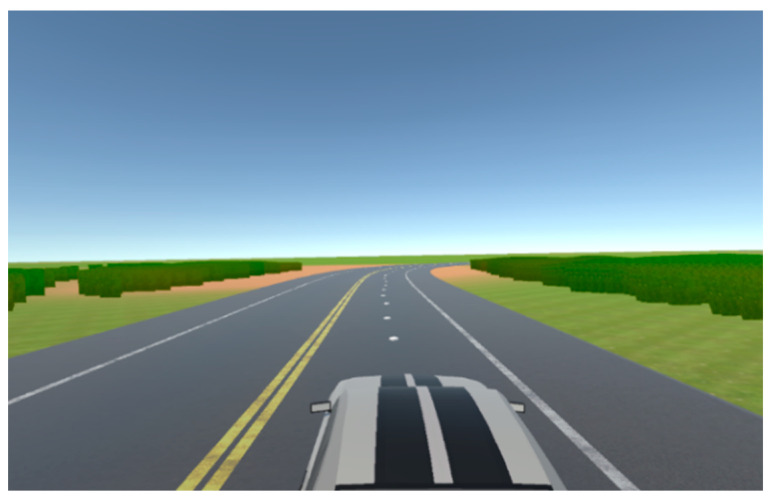
Depiction of vehicle following set waypoints path.

Given that the proposed automatic data collection model assimilates the driver’s driving capabilities, the challenge of manually driving the virtual car using a PS4 controller hinders large-scale data collection. To address this issue, a solution for automating driving in the sandbox environment was devised involving the implementation of waypoints strategically positioned within the lane. These waypoints delineate the optimal path for the vehicle, thereby facilitating the generation of high-quality training samples. As the virtual vehicle traverses the road, it continuously detects the closest waypoint based on its current position. Subsequently, leveraging its position relative to the waypoint, the vehicle calculates the optimal steering angle required for alignment. Upon reaching a designated waypoint, it is discarded, and the subsequent waypoint in sequence becomes the new target for steering determination, thereby perpetuating the iterative process. Figure 6 shows the vehicle following the set waypoints path.

The vehicle adeptly computes the optimal steering angle required for navigation; however, the instantaneous transition from, for instance, 10 degrees to 30 degrees fails to faithfully replicate human driving behavior. To mitigate this abruptness, a smoother transition is achieved by linearly interpolating between the current steering angle of the vehicle and the destination angle using the following Equation (1):

(1) ∅=θ+(φ−θ)×v

where ∅ is the interpolated angle, *θ* is the current angle, *φ* is the end angle, and *v* is the speed of the vehicle.

Furthermore, the interpolation process accounts for the vehicle’s speed, ensuring that the transition occurs at an appropriate pace, thereby enhancing the fidelity of the driving simulation. The sandbox driving scenarios can be diversified by generating additional car objects on the same road using Unity3D AI features to drive following the same set of waypoints as the main agent [41]. The AI functions available in Unity3D offer a degree of autonomy to these additional car objects. There are two main strategies for adapting the navigation of the main agent in multi-car scenarios, dictated by whether the agent is environment-agnostic or environment-aware. In the environment-agnostic case, the agent can be equipped with virtual sensors (such as the camera used in the current implementation, but also radar-type sensors that monitor and measure proximity) and with a control unit that adapts the maneuvers based on the information available on the dynamic scene. In the environment-aware case where the main agent is part of multiple vehicle platoons, the sandbox environment can be adapted to include additional parameters that can be made available to the main agent, such as the coordinates of other vehicles (modeling vehicle-to-vehicle communications), road geometry, and lane position (modeling vehicle-to-everything (v2x) communications). This would augment the information available for training the navigation. The control unit of the vehicle can be based on the principles outlined in [42,43] and allow the generation of training samples in the virtual sandbox.

### 3.2. Data Balancing

**Figure 7:**
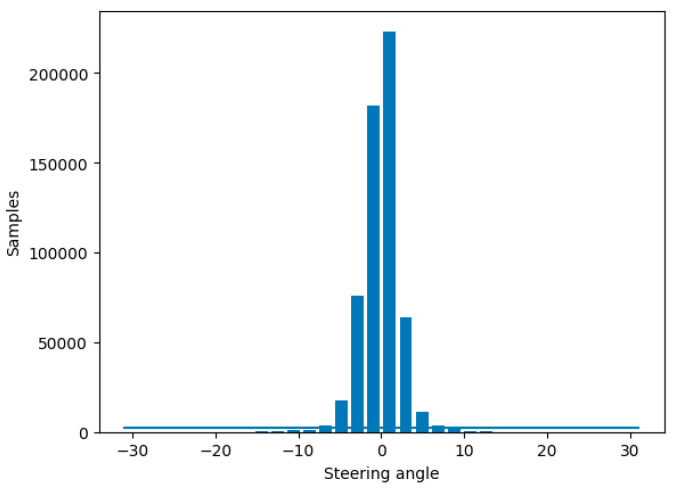
The distribution of steering angles from the collected dataset.

Each sample of the collected data includes four columns: the extracted image paths from the left, front, and right cameras; and an entry for the steering angle associated to the captured snapshots at the current point of driving. Since there are not many turns on country roads, most of the data collected would come from samples where the steering angle is close to 0°, meaning the automobile is traveling straight. A model trained on the distribution shown in Figure 7 would become heavily biased toward driving straight and would struggle to perform effectively.

To address this problem and improve the data balance, samples must be excluded at levels over a certain threshold. However, samples must be rejected at random locations on the track where the automobile was located because if a large section is removed, the dataset would lose a large chunk of the track and result in a road texture not being introduced to the model at training time. The samples rejected were for steering angles of ±2.5°, which were considered a good approximation of straight road driving.

**Figure 8:**
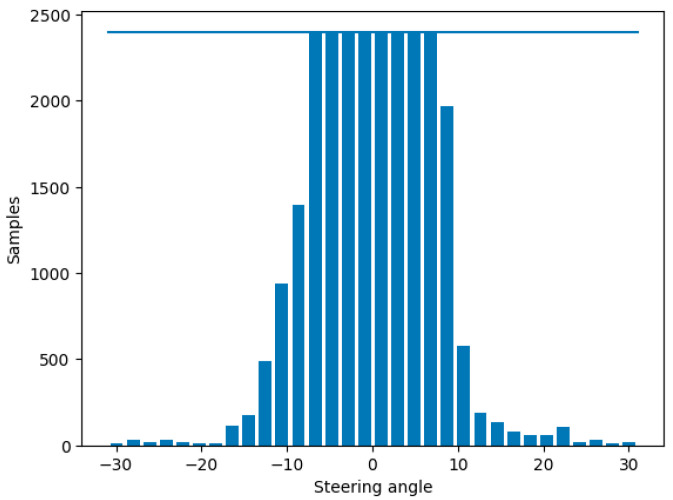
Distribution of steering angles in the dataset after conducting the balancing using threshold method.

The total size of the dataset is 587,547; however, the threshold of sample rejection is set quite low (400 per bin) given how many samples there are of the vehicle driving mostly straight, which would cause the model to become biased towards driving straight. During this process 582,299 are removed, leaving only 5248 samples to be split into training and validation sets (as shown in Table 1). The majority of the samples are rejected; however, this step is required to ensure a balanced dataset, depicted in Figure 8.

### 3.3. Data Augmentation & Preprocessing

**Figure 9:**
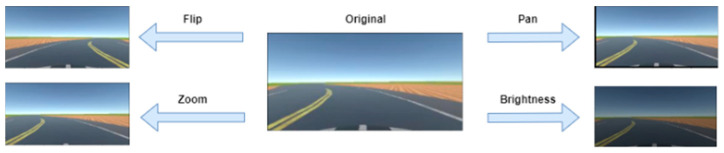
Overview of the data augmentation techniques applied.

Data augmentation methods were used to increase the dataset: flip, zoom, changing the brightness, and panning, as depicted in Figure 9.

**Figure 10:**
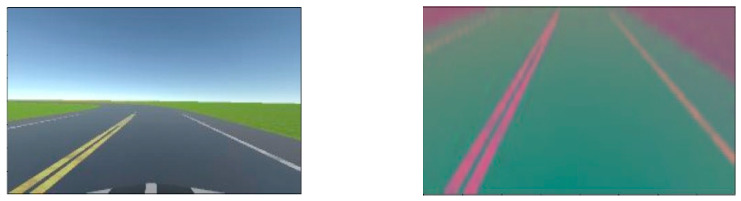
Road view before and after pre-processing.

When the model is being trained, there is a 50% chance one of the augmentation methods illustrated in Figure 9 is applied to an image during training. The type of augmentation is randomly selected; as such the augmentation techniques are equally distributed in the training set (25% probability of one of the following augmentations being applied: flipping, zooming, brightness change, panning). Gaussian blur is applied to the images to reduce noise and help the model extract features more easily. Next, the images are resized to 200 × 66, this results in faster computations since smaller images are easier to work with. Finally, the images are normalized to ensure each pixel has a similar distribution. Figure 10 illustrates a road view before and after pre-processing.

### 3.4. Proposed CNN Model

The CNN model architecture developed in [44] was considered a base for the proposed current model. The primary objective during training is to optimize the network’s weights to minimize the loss function, resulting in a more accurate model for predicting the required steering angle to keep the autonomous vehicle (AV) within its lane on the road.

**Figure 11:**
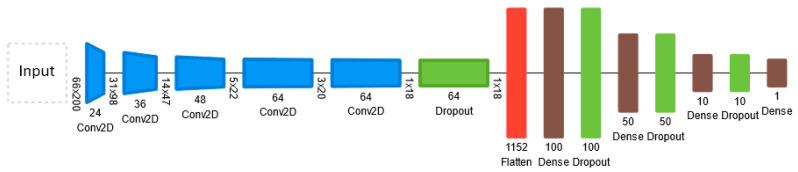
CNN architecture.

The proposed CNN architecture comprises 5 convolutional layers and 4 fully connected layers (Figure 11). In this current implementation, we have omitted the normalization layer, as data normalization is conducted prior to training. The convolutional layer of this proposed model is the key layer that absorbs the gathered images when independently driving and uses a kernel-based feature detector to extract features from them. The kernel is modeled as a 2D matrix that is applied to a specific region in the matrix, with the result of the dot product calculation being sent into an output array. The kernel is moved to the next location in the image by a 2 × 2 stride after the dot product calculation, and this process is repeated until the feature detector has gone through the entire image. The output of the convolutional layers is fed into an activation function to introduce non-linearity. The activation function used is exponential linear unit (ELU) which is very similar to the most commonly used activation function ReLU, but allows for negative values, which is an essential feature for this use case as it permits coding steering angles to the left.

(2) $$f\left(z\right)=\{\begin{array}{rr} z,\;\;\;\; & z>0\\ \alpha \left(e^{z}-1\right),\;\;\;\; & z\leq 0 \end{array}$$

Convolutional layers are not densely connected which allows for more flexibility when learning and moreover, the number of weights in the layers is a lot smaller. These characteristics allow the convolutional layers to extract features from the image [45]. The extracted feature from the convolutional layer is then passed through a flattened layer which converts the resultant 2D matrix into a 1D continuous vector of extracted features. Introducing FC layers allows the CNN model to mix signals and exchange information between each dimension since every neuron is connected to the next one in the next layer. The result from each layer is then passed through the ELU activation function and the loss at each iteration is calculated.

After that, the CNN model was fed into the label and the prediction to the loss function during training to measure how well the model is predicting the labels. The main aim was for CNN to use this performance outcome to modify its hyper-parameters so that the loss function’s output closely resembles the provided target (the average loss). The loss function that is used in the architecture is mean squared error (MSE) by applying the following equation, In the formula, where n represents the number of samples, y denotes the observed steering angle, and $\hat{y}$ represents the predicted angle, the mean square error is calculated by summing the squared differences between observed and predicted values.

(3) $$MSE=\frac{1}{n}\overset{n}{\underset{i=0}{\sum }}{\left(y^{i}-{\hat{y}}^{i}\right)}^{2}$$

The mean squared error (MSE) function calculates the average of the squared differences between predictions and labels. This function is particularly sensitive to outliers, meaning that if the prediction significantly deviates from the label, it will result in a higher loss value. Additionally, the MSE function possesses a well-defined global minimum, making it compatible with optimization techniques like gradient descent, which we will also incorporate into the neural network architecture.

In the realm of machine learning, optimization algorithms play a pivotal role in reducing the loss function at each training iteration, thereby enhancing the model’s accuracy. In the context of convolutional neural networks (CNNs), this research employed the ADAM [46] optimization method which is commonly employed as an alternative to traditional stochastic gradient descent techniques. It serves the purpose of dynamically adjusting the model’s hyperparameters throughout the training process to minimize the loss function. The hyperparameters can be iteratively adjusted and fine-tuned by further training on new pre-processed balanced data samples beyond the experiment reported here.

The data underwent preprocessing before being divided into training and validation sets using an 80/20 split ratio which contains 13,684 training samples and 3422 validation samples. The hyperparameters used to train the CNN model (epochs and batch sizes) were adjusted through trial and error and finally, a batch size of 128 was used in 50 epochs. At each iteration, the batches consisting of images and steering angles are randomly augmented with the techniques stated above in order to artificially inflate the data, so the model has more exposure to learning features.

By strategically placing dropout layers immediately after the flattening layer and as the final layer of the model, it was observed through the experiment that overfitting could be mitigated, particularly given the size of the training dataset. This approach aims to regularize the model during training by randomly dropping a fraction of the connections between neurons, thus reducing the risk of the model memorizing noise or specific patterns in the training data that may not generalize well to unseen data.

### 3.5. Real-World Driving Evaluation Data

For model evaluation, a testing dataset sourced from real-world driving was obtained from Comma.ai [47], which encompassed 45 GB of highway driving footage, capturing videos and documenting the wheels’ turning angle at each timestamp, along with other relevant properties. A notable distinction from our sandbox data lay in the fact that the steering angle of the tires rather than that of the wheel was recorded in this dataset.

To be able to use this dataset to test our model, we identified the vehicle used by Comma.ai during data collection, specifically an Acura ILX 2016 model, and used the specifications of this vehicle to map these details to a corresponding vehicle model within Unity3D. Two adjustments were necessary to ensure compatibility and accuracy between the real-world vehicle and its virtual counterpart. Upon recognizing that the steering wheel’s rotation did not directly correspond to the turning of the wheels at a 1:1 ratio, this paper consulted Ackermann’s steering geometry principles [48] to determine a ratio of 15.3:1 for the real car. By using the curb-to-curb distance and wheelbase distance of the actual vehicle, the maximum wheel turning angle was calculated and subsequently, the mapping between the steering wheel and wheel turning angles was adjusted to ensure accurate simulation.

## 4. Results

The convolutional layers of the model are responsible for extracting features from the input snapshots. These features can be visualized after extracting the internal state of the model. The contrast between the lanes and their boundaries helps the model detect the edges and predict the needed steering angle to keep between the boundaries. To assess the model’s performance, several prerequisites must be met. Firstly, a testing dataset is assembled by creating an automatic data collection process within Unity3D. In this virtual environment, the vehicle is automatically driven, and snapshots of the road are collected, akin to the process employed in gathering the training dataset.

Finally, validation is performed with real-world data, serving to assess the model’s resilience and adaptability to various real-world conditions. The performance of the model was evaluated on real-world highway driving data using the Comma.ai dataset [47]. The positioning of the vehicle within the lane must be ascertained, for which we use the autonomy metric proposed in [44].

**Figure 12:**
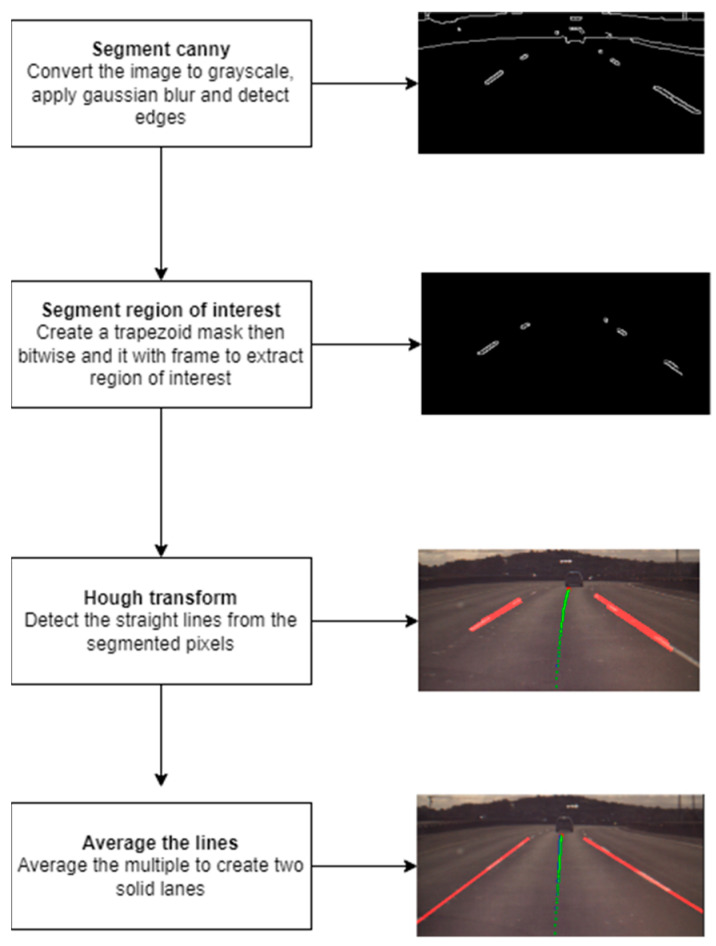
Lane detection workflow. (Bottom image at the right hand side: red lines represent detected lane boundaries, the blue line shows the road’s central alignment, and the green line indicates the vehicle’s projected path).

Before precise vehicle localization can be achieved, it is necessary to identify lane boundaries from real-world driving data. The lane detection workflow depicted in Figure 12 works by applying Canny edge detection on each frame of the image in order to segment the lane boundaries. First, the video frame is converted to grayscale and Gaussian blur is applied to reduce the noise before Canny edge detection is applied. Canny would segment the edges of objects that fall between the set white threshold of 50 to 150.

The edge detection will detect all edges in the frame that fall within that threshold; however, we are only interested in the lane boundaries. To reduce the noise and apply the filter only on a region of interest, a trapezoid would specify the region of interest we want to apply the filter. This region of interest encloses the lane boundaries the vehicle is traveling on and detects the edges of the lane. A bitwise AND operator can be applied between the mask created and the Canny filtered frame to segment only the lane boundaries from the image.

After the pixels of interest are selected, a Hough transform is applied that transforms these pixels into multiple straight lines to form the boundaries of the lane.

Since the Hough transform produces multiple lines but we are only interested in the left and right boundaries of the lane, all the lines are passed to a function which averages them by taking the into account the slope and y-intercept of each the lines turning them into solid lines which form the left and right boundaries of the lane.

To ascertain the centrality position precision of the car within the lane, the width of the lane is first determined by computing the distance between the left and right boundaries. Subsequently, the distance from the predicted position of the center of the car to the middle point of the lane is measured. The vehicle’s position precision is calculated using the formula:

(4) $$centrality\;precision=\{\begin{array}{rr} -\frac{2}{l-w}d+1,\;\; & d<\left(l-w\right)/2\\ 0,\;\; & d\geq \left(l-w\right)/2 \end{array}$$

where *w* is the width of the car, *l* is the width of the lane and *d* is the distance between the midpoint of the vehicle and the center point of the lane. The resulting number is multiplied by 100 to attain a percentage. As such, a precision value of 100% signifies perfect alignment at the lane center (d = 0), while 0% indicates that the car crosses either the left or right lane boundaries (d > (*l −* w)/2). This formulation enables the calculation of the centrality precision, facilitating the determination of instances when the vehicle is projected to deviate from the center of the lane.

We use the position centrality precision as a metric for assessing lane-keeping performance and potential deviations from desired trajectories in the broader context of autonomous driving systems. The resultant data from testing have been split into distinct positional buckets delineated by centrality precision ranges: [0–25%, 25–50%, 50–75%, 75–100%], representing the centrality of the vehicle within the lane at each frame. Additionally, a supplementary category, “Outside of lane boundaries”, has been introduced to capture instances where the vehicle’s predicted position falls outside the detected lane boundaries.

To further ascertain the performance of the proposed deep learning-based lane detection and steering model, we use the autonomy metric proposed by [44], which measures how often a human must intervene to keep the self-driving car on the lane.

If the car strays away from the center of the lane more than a set threshold, it would be intervened by a person and repositioned accordingly. Repositioning the car and resetting the self-driving predictor takes approximately 6 s.

The autonomy percentage of the vehicle is calculated by subtracting the number of interventions (*N*) multiplied by the time it takes for a vehicle to be recentered by a human (6 s) divided by the total time of the experiment in seconds. Then multiplied by 100 to attain a percentage.

(5) $$autonomy\;=\;\left(1\;-\;\frac{N\times 6}{time}\right)\times 100$$

In our testing environment, intervention by a person to recenter the car is not permitted; however, the autonomy metric can be calculated by considering when human intervention is required to correct the course of the car, i.e., when a set deviation from centrality occurs. Our testing experiment has been adapted to indicate when the model needs correction. To calculate the autonomy of the car in our experiment, we set the condition that a human intervention is required when the centrality precision drops below 50%.

We considered two evaluation scenarios using appropriate real driving footage from the Comma.ai dataset: (i) driving on mostly straight roads; (ii) driving on roads with sharp turns.

**Figure 13:**
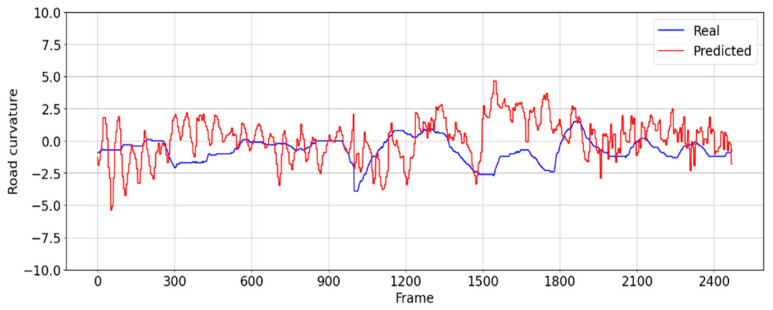
Comparison between the steering angle the real driver (blue) and the predicted steering angle (red) generated by the proposed CNN model from the road footage for evaluation scenario (i).

In the evaluation scenario (i), a roadway characterized by minimal ambient noise was selected, ensuring conditions conducive to stable lane boundary detection. Specifically, this test footage presented a driving scenario devoid of obstructions such as vehicles directly in front of the testing vehicle, with lane changes enacted to maintain consistent lane boundary detection throughout the evaluation process. The steering angle of the real driver varies from 2.5 degrees to −2.5 degrees from the center. Figure 13 shows that the CNN model is able to follow a similar steering pattern, with an average error from ground truth of 1.6 degrees.

**Figure 14:**
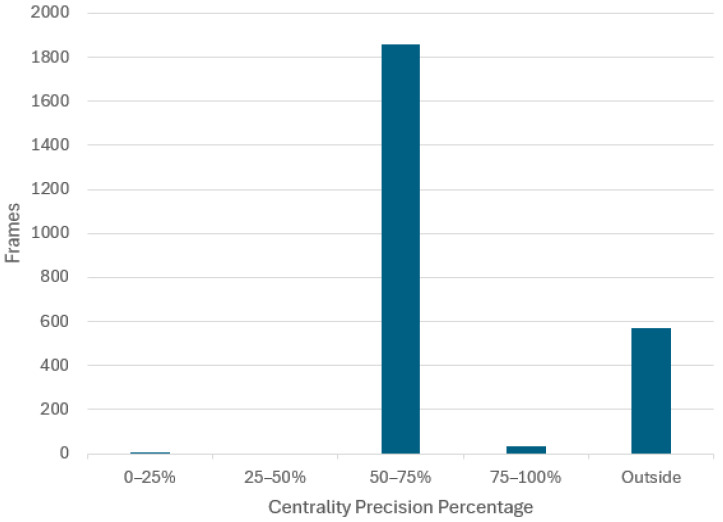
The histogram of position centrality precision generated by the proposed CNN model from real-world driving footage (evaluation scenario (i)).

The distribution of frames across the defined centrality precision buckets when the CNN model is employed to drive in this scenario is presented in Figure 14 and Table 2.

This assessment scenario comprises a total of 2471 frames, of which there are 575 frames where the vehicle had a centrality precision below 50%. Since the footage is recorded at 100 frames per second, this means that it will take 600 frames for the vehicle to be repositioned back to the center lane, assuming the 6 s intervention time. It yields that there are *N* = 575/600 = 0.96 times when the vehicle is off center, which gives 0.96 interventions for the 24.7 s video. Using Equation (5), this results in an autonomy of 77%:

(6) $$autonomy\;=\;\left(1\;-\;\left(\frac{0.96\;\times \;6}{24.7}\right)\right)\;\times \;100\;=77\%$$

**Figure 15:**
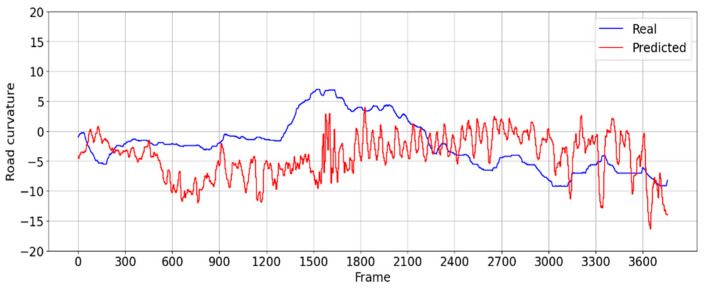
Comparison between the steering angle the real driver (blue) and the predicted steering angle (red) generated by the proposed CNN model from the road footage for evaluation scenario (ii).

In the more challenging evaluation scenario (ii), a road with sharper turns is used, where the steering angle of the real drivers varies from 6 to −9 degrees away from the center. Figure 15 shows in comparison the steering angle of the CNN driver versus the steering angle of the real driver, exhibiting an average error of 5 degrees.

**Figure 16:**
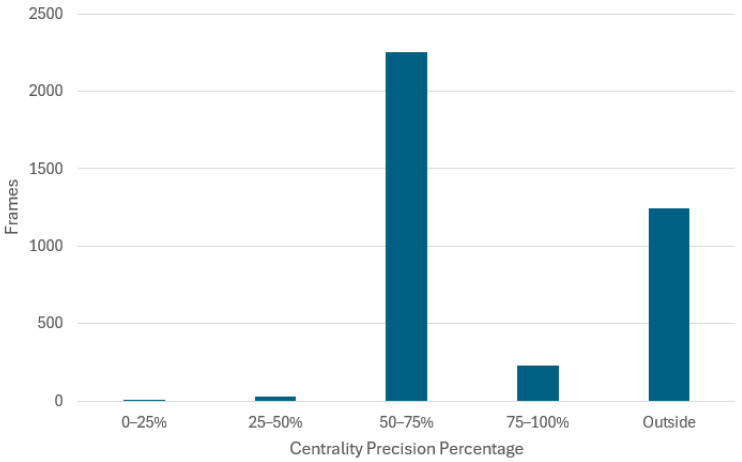
The histogram of position centrality precision generated by the proposed CNN model from real-world driving footage (evaluation scenario (ii)).

For scenario (ii), the distribution of frames across the defined centrality precision buckets when the CNN model is employed to drive in this scenario is presented in Figure 16 and Table 3.

Of the total of 3759 frames comprised in scenario (ii), there are 1277 frames where the vehicle had a centrality precision below 50%. Similar calculations to those used for scenario (i) yield that there are *N* = 1277/600 = 2.13 times when the vehicle is off center, i.e., 2.13 human interventions necessary for the 37.6 s video. Using Equation (5), this results in an autonomy of 66%:

(7) $$autonomy\;=\;\left(1\;-\;\left(\frac{2.13\;\times \;6}{37.6}\right)\right)\;\times \;100\;=66\%$$

## 5. Conclusions

In conclusion, the end-to-end framework proposed offers a systematic approach to mitigating costs and environmental impact associated with data collection and training deep learning steering angle predictors. By automating driving sample collection in a controlled sandbox virtual environment, the potential for human error is significantly reduced, leading to cost and time savings while minimizing environmental footprint. Furthermore, the versatility of the methodology allows for the creation of new training chunks, facilitating targeted improvement in areas where models exhibit weaknesses. We have demonstrated the value of the end-to-end customizable framework for training an automated lane detection and steering system by implementing a behavioral driving model where lane markings are not necessary to detect the lane and where frame annotation with corresponding steering angle is automated. Furthermore, using this framework, a CNN-based autonomous driving model has been developed, enabling vehicles to stay within designated lanes with an autonomy of 77% in low challenge road conditions and 66% on roads with sharper turns.

While challenges persist, particularly on roads with sharper turns commonly found in urban environments and ambiguous or absent lane markings, the proposed framework opens the way for an iterative approach to evaluation and refinement, which is crucial for enhancing the model’s reliability and generalization capabilities.

In summary, the proposed method represents a significant step forward in developing cost-effective and environmentally sustainable solutions for training deep learning steering angle predictors. By combining automated sample collection, flexibility in training data utilization, and thorough performance evaluation in real-world conditions, the method contributes to the advancement of autonomous driving technologies while minimizing associated costs and environmental impact.

## Figures and Tables

**Table 1:** The collected samples.

Collected	Rejected	Training/Validation
587,547	582,299	5248

**Table 2:** The distribution of frames across the defined centrality precision buckets for evaluation scenario (i).

0–25%	25–50%	50–75%	75–100%	Outside
5	0	1860	36	570

**Table 3:** The distribution of frames across the defined centrality precision buckets for evaluation scenario (ii).

0–25%	25–50%	50–75%	75–100%	Outside
5	30	2255	227	1242

## Data Availability

The data presented in this study are available on request from the corresponding author.
